# Towards implementing exercise into the prostate cancer care pathway: development of a theory and evidence-based intervention to train community-based exercise professionals to support change in patient exercise behaviour (The STAMINA trial)

**DOI:** 10.1186/s12913-021-06275-w

**Published:** 2021-03-22

**Authors:** Sophie Reale, Rebecca R. Turner, Eileen Sutton, Stephanie J. C. Taylor, Liam Bourke, Dylan Morrissey, Janet Brown, Derek J. Rosario, Liz Steed

**Affiliations:** 1 Allied Health Professionals, Radiotherapy and Oncology, Sheffield Hallam University, Sheffield, UK; 2 Population Health Sciences, University of Bristol, Bristol, UK; 3 Institute for Population Health Sciences, Queen Mary University of London, London, UK; 4 Sports and Exercise Medicine, William Harvey Research Institute, School of Medicine and Dentistry, Queen Mary University of London, London, UK; 5 Department of Oncology and Metabolism, University of Sheffield, Sheffield, UK; 6 Department of Urology, Sheffield Teaching Hospitals, Sheffield, UK

**Keywords:** Prostate cancer, Androgen deprivation therapy, Exercise, Exercise professionals, Behaviour change, Intervention development, Behaviour change wheel, Person based approach, Medical Research Council, Patient and public involvement

## Abstract

**Background:**

The National Institute for Health and Care Excellence (NICE) recommend that men on androgen deprivation therapy (ADT) for prostate cancer should receive supervised exercise to manage the side-effects of treatment. However, these recommendations are rarely implemented into practice. Community-based exercise professionals (CBEPs) represent an important target group to deliver the recommendations nationally, yet their standard training does not address the core competencies required to work with clinical populations, highlighting a need for further professional training. This paper describes the development of a training package to support CBEPs to deliver NICE recommendations.

**Methods:**

Development of the intervention was guided by the Medical Research Council guidance for complex interventions and the Behaviour Change Wheel. In step one, target behaviours, together with their barriers and facilitators were identified from a literature review and focus groups with CBEPs (*n* = 22) and men on androgen deprivation therapy (*n* = 26). Focus group outputs were mapped onto the Theoretical Domains Framework (TDF) to identify theoretical constructs for change. In step two, behaviour change techniques and their mode of delivery were selected based on psychological theories and evidence to inform intervention content. In step three, the intervention was refined following delivery and subsequent feedback from intervention recipients and stakeholders.

**Results:**

Six modifiable CBEPs target behaviours were identified to support the delivery of the NICE recommendations. Nine domains of the TDF were identified as key determinants of change, including: improving knowledge and skills and changing beliefs about consequences. To target the domains, we included 20 BCTs across 8 training modules and took a blended learning approach to accommodate different learning styles and preferences. Following test delivery to 11 CBEPs and feedback from 28 stakeholders, the training package was refined.

**Conclusion:**

Established intervention development approaches provided a structured and transparent guide to intervention development. A training package for CBEPs was developed and should increase trust amongst patients and health care professionals when implementing exercise into prostate cancer care. Furthermore, if proven effective, the development and approach taken may provide a blueprint for replication in other clinical populations where exercise has proven efficacy but is insufficiently implemented.

**Supplementary Information:**

The online version contains supplementary material available at 10.1186/s12913-021-06275-w.

## Background

Prostate cancer is common worldwide, totalling approximately 1.3 million new cases in men in 2018 [1]. More specifically, prostate cancer is the most common cancer diagnosis in men in the UK, with 41,201 new cases in 2017 [2]. Approximately half of men diagnosed with prostate cancer will undergo androgen deprivation therapy (ADT), also known as medical castration [3]. This is an ongoing treatment administered via injection, tablet or surgery. Whilst ADT is effective in treating prostate cancer, persistent adverse effects are common and can be debilitating thus impacting on overall quality of life (QoL) (e.g. fatigue, reduced muscle mass, loss of bone mineral density, hot flushes, and sexual dysfunction) [4].

Numerous strategies to manage the side effects of ADT have been described and tested in practise [5]. However, to date the only evidence-based treatment to demonstrate clinically meaningful improvements to fatigue and disease specific QoL, are non-pharmacological interventions, predominantly exercise training [6, 7]. As such, the National Institute for Health and Care Excellence (NICE; NG131 1.4.19) include in the guidelines for locally advanced and advanced prostate cancer a recommendation that men on ADT should be offered supervised, aerobic and resistance exercise twice weekly for 12 weeks [8]. However, these recommendations are seldom integrated into practise [9] due to several reported barriers from healthcare professionals (HCPs), including a lack of exercise services to refer men to [10] and limited awareness of clinical exercise recommendations [11], thus adding to unmet needs reported by men with prostate cancer [12]. Furthermore, men on ADT who participated in a 12-month supervised exercise programme believe community gyms are easily accessible and a location they look forward to attending [13].

A genuine opportunity exists to improve the QoL of men with prostate cancer on ADT, with exercise professionals supporting the delivery of recommendations nationally (i.e. NG131 1.4.19). More specifically, findings from our earlier work, including interviews with a diverse range of healthcare professionals working in the NHS prostate cancer care and exercise referral pathway highlight that HCPs and allied health professionals, (including physiotherapists), do not perceive delivering NICE recommendations (i.e. NG131 1.4.19) as part of their role [9]. Instead, community-based exercise professionals (CBEPs) (i.e., physiologists, physiotherapists and personal trainers based within the leisure industry) are deemed more suitable. For example, the role of CBEPs is to design and deliver safe and effective exercise programmes. Their success in this role has been demonstrated in previous exercise trials e.g. [14] and is in line with regulation by the Chartered Institute for the Management of Sport and Physical Activity (CIMSPA) [15]. Therefore, we intend a service level change in the prostate cancer care pathway by integrating two currently distinct sectors (the leisure industry and the NHS). We aim to explore the implementation of NICE recommendations into practice, as part of an ongoing research project; STAMINA (Supported exercise TrAining for Men wIth prostate caNcer on Androgen deprivation therapy).

However, integration of these two sectors is not without challenge. Interviews with HCPs in the prostate cancer care pathway have highlighted concerns regarding patient safety when exercising in the community due to a lack of confidence in CBEP’s skills to deliver a safe, and disease specific, exercise programme [9]. Similarly, men on ADT emphasise the need for exercise to be supervised by a professional and tailored to meet their individual needs [9]. These beliefs are possibly reflective of limited HCP and patient knowledge of the training CBEPs receive, the typically short duration and/ or limited regulation for personal training qualification [16]; the variation in CBEPs behaviour change and communication skills, and limited CBEPs knowledge related to prostate cancer. Evidence suggests standard exercise professional qualifications do not address the core competencies of working with clinical populations highlighting a need for further appropriate professional training to ensure safety [16].

This paper describes the methods used and outcomes realised from the development and refinement of a theory and evidence-based intervention (as part of the STAMINA programme) to facilitate CBEPs to support men on ADT for prostate cancer to initiate, and further maintain, exercise behaviour. The structure was guided by formalised intervention development approaches: the UK Medical Research Council (MRC) guidance for the development and evaluation of complex interventions, emphasising the importance of drawing on evidence and theory and specifying key steps in the intervention development process [17], and the Behaviour Change Wheel (BCW) [18] complemented by elements of the Person-Based Approach [19], as has been used previously [20]. The BCW is a guide to intervention development informed by theory and based on the synthesis of 19 behaviour change frameworks [18]. The Person-Based Approach complements the BCW by focussing primarily on users’ perspective and the context of an intervention to enhance acceptability and the likely engagement with, and effectiveness of, an intervention [19].

The aim of this research was to develop a theory and evidence-based training package for CBEPs to support the delivery of the NICE recommendations NG131 1.4.19, prior to full effectiveness and implementation evaluation in a planned randomised controlled trial. In parallel, training for HCPs to provide exercise recommendation and support was developed after further professional training requirements were identified as part of the STAMINA programme (methods reported elsewhere; *under review*).

## Methods

**Fig. 1:**
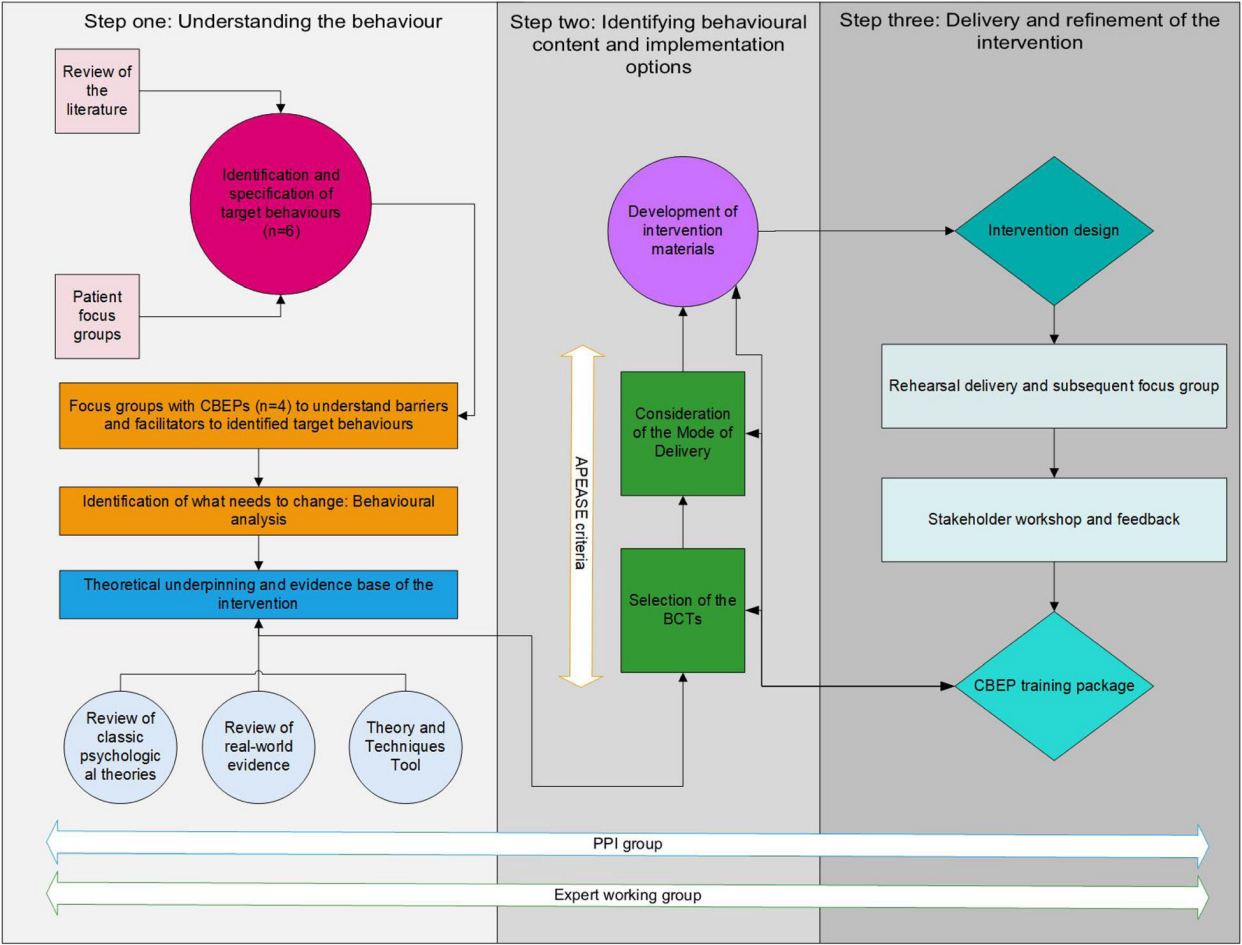
Iterative intervention development process of a training package for community-based exercise professionals (CBEPs)

The intervention was developed in accordance with the MRC guidance for the development of complex interventions [17, 21] and took a theory and evidence based approach. The BCW [18] in conjunction with elements of the Person-Based Approach [19] provided a systematic guide to intervention development, incorporating user perspectives, with the aim of changing CBEPs behaviour. The intervention was refined through an iterative and dynamic process based on evidence, theory and feedback from intervention recipients, patient and public involvement (PPI) members, stakeholders and an expert working group with expertise in: behaviour change (SR, LS, RT), complex intervention development (ST), cancer research (LB, DR), urology (DR) and qualitative methodologies (ES) (Fig. 1).

Regulatory and ethical approvals, in accordance with the Helsinki Declaration, were sought prior to the commencement of research activities from Sheffield Hallam University (Reference: ER10748795) and the NHS (REC reference: 18/NW/0738 / IRAS project ID: 254343). Written informed consent was collected from all participations prior to research activity.

### Step one: understanding the behaviour

To develop a complex intervention centred on supporting patient behaviour, we firstly sought to understand patient needs i.e. what support do men on ADT for prostate cancer need to exercise. We then identified how to train CBEPs to deliver the required support to men on ADT, as detailed below.

#### Identification and selection of target behaviours

A list of potential target behaviours for patients and CBEPs were generated by reviewing i) existing literature examining the effectiveness of interventions aimed at exercise initiation and maintenance of cancer survivors and the role of exercise professionals [22] and ii) results of 5 focus groups with men on ADT for prostate cancer (*n* = 26), exploring their experiences of physical activity and beliefs about participating in a structured exercise programme (methods reported elsewhere [9]).

The comprehensive list of behaviours was reviewed by the expert working group for replication or cross-over between behaviours. Each behaviour was then coded according to defined criteria from the BCW [18], to identify behaviours that should be targeted in the intervention: a) the likely impact of change, b) the likely ease of change, c) the centrality of the behaviour (e.g. the likelihood of changing one behaviour having an impact on another behaviour) and d) the ease of measurement. Coding was completed by an individual researcher (SR) and verified through discussion with the expert working group including review by a behaviour change expert (LS).

#### Specification of target behaviours

Next, the target behaviours were specified in behavioural terms by the expert working group, and reviewed by the PPI group (7 men with prostate cancer and 4 family members); *who* needs to deliver the behaviour, *what* does a person need to do differently, *when* will it happen and *where* will it take place. To consider dose we also considered *how often* the behaviours are required and *with whom*.

#### Identifying what needs to change

To further develop our understanding of how CBEPs may support men on ADT to exercise, barriers to, and enablers of the patient target behaviour were identified in the previously reported focus groups [9]. Men were recruited from two urology out-patient departments in South Yorkshire and Derbyshire. All eligible men presenting during routine follow-up care, prostate cancer support groups or via poster advertisement were approached for participation.

Similarly, barriers and facilitators of the CBEPs target behaviours that could be addressed in a training programme were identified from four focus groups. CBEPs were identified from four Nuffield Health fitness and wellbeing gyms using purposive sampling to ensure inclusion of the target population (community-based personal trainers, physiologists, and fitness managers) and relevant stakeholders (gym general managers and operational managers). Study documents were sent via the research team to eligible Nuffield Health employees. Those who provided written consent were enrolled into the study. Each focus group was led by an experienced facilitator (ES, LS, SR) and the topic guide, reviewed by the PPI group, was designed to explore the 14 domains of the Theoretical Domains Framework (TDF) version 2.0 [23] (Additional file 1). The TDF is an integrative framework originally based on the synthesis of 128 theoretical constructs from 33 theories of behaviour change [24] to understand behaviour at an individual level and support the development of implementation interventions [25]. Furthermore, CBEPs were probed on their preferred mode of delivery of the professional training programme (e.g. online versus face-to-face).

Responses from the focus groups were mapped onto the TDF to identify domains through which change may occur. Focus group transcripts were analysed deductively supported by the use of NVivo [26]; the TDF was used to generate the framework for content analysis (SR) and coding specifically identified the type of professional (or patient) who provided the information and the type of professional (or patient) the information was related to (e.g. themselves, a different exercise professional, a HCP or patient). The lead researcher (SR) systematically went through each transcript, coding according to the framework. Text was attributed to more than one domain where applicable. Text relating only to the target behaviours were coded for relevance [25]. Subsequently, 25% of coding was double coded with a second independent reviewer (LS), to develop the coding framework as described above (i.e. who provided the information and the type of professional the information was related to). The coders were in 85% agreement, and following discussion, all discrepancies were resolved.

#### Theoretical underpinning and evidence base

Following the behavioural analysis, psychological theories from the field of behaviour change that related to the identified constructs within the TDF were then reviewed to consider how best to apply them to the current context and advance our understanding of the likely mechanisms of change. In line with MRC guidance for the development and evaluation of complex interventions, a logic model was developed to present the proposed mechanisms of change of the intervention [27].

### Step two: identifying behavioural content and implementation options

#### Behaviour change techniques

Having identified which theoretical constructs required change to achieve both the patient and CBEPs target behaviours, content for the intervention was developed and guided by the inclusion of behaviour change techniques (BCTs). BCTs are defined as observable, replicable, and irreducible components of behaviour change interventions [18]. We used the labels and detailed definitions of BCTs as those included in the BCT Taxonomy version 1 (BCTTv1) [28].

BCTs to be delivered by the CBEPs to support long-term exercise behaviour were identified from previously drawn links between constructs of the TDF [18], mechanisms of action [29] and previous literature exploring BCTs for promoting exercise behaviour in people living with and beyond cancer [22]. Context-based decisions for each BCT that was identified as likely to be effective (i.e. most frequently used with the selected TDF domains), and supported by evidence, was made in relation to its affordability, practicability, effectiveness and cost-effectiveness, acceptability, side effects and safety and equity considerations (APEASE criteria) [18].

Similarly, inclusion of BCTs for the delivery of the exercise professional training were guided by the CBEPs target behaviours and theoretical constructs requiring change. BCTs were identified from previously drawn links between constructs of the TDF [18] and mechanisms of action [29]. BCTs that were identified as effective (i.e. most frequently used with the selected TDF domains) were reviewed against the APEASE criteria and CBEPs focus group findings to determine their suitability in the intervention [18].

#### Mode of delivery

Once the BCTs had been identified, the behavioural content was then developed, specifying the mode of delivery. A range of feasible modes of delivering the selected BCTs were considered and reviewed against the APEASE criteria [18] to identify content to be delivered via traditional face-to-face approaches and online learning technologies to promote active and self-directed learning [30].

Furthermore, intervention materials including patient booklets and professional training worksheets were created alongside a training manual for CBEPs and a facilitator manual for the training providers.

### Step three: delivery and refinement of the intervention

Modelling components of a complex intervention prior to a full-scale study provides important information about the design of the intervention [17]. We sought to operationalise the CBEPs training package during an iterative process, delivering the intervention face-to-face to CBEPs (*n* = 11) and stakeholders (*n* = 28) between February and June 2019, for feedback and refinement of the intervention.

#### Rehearsal delivery and subsequent focus groups

The exercise professional training package was delivered to CBEPs who were purposively sampled to ensure a diverse range of target users (e.g. gender, job role and previous experience) [19]. Feedback on the content, format, location and delivery of the intervention were collected immediately post intervention, via an audio recorded focus group with an independent researcher (RT). The topic guide was based on Kirkpatrick’s Four Level Training Evaluation Model [31] (Additional file 1) and considers reaction - concerned with understanding how participants feel about the training programme, learning - the extent the attendees have learnt something new such as skills, behaviour - the extent training is put in to practice and results that are obtained as a result of the intervention. Focus group data were analysed in NVivo [26] following Braun and Clarke’s six step process of thematic analysis [32]. Suggestions for change were highlighted from the key themes and rated against MoSCoW criteria (Must have, should have, could have, would like) for prioritising change [33]. Modifications were made if they were considered likely to impact on behaviour change, uncontroversial and easy or recommended by multiple participants.

#### Stakeholder workshop and feedback

Following the rehearsal delivery, the exercise professional training package and patient intervention materials were presented at a one-day stakeholder workshop to enhance the future implementation of the designed intervention. Participants were identified via existing clinical, professional and patient networks, national charity representatives and our PPI group. The stakeholder workshop ran in the format of presentations from the research team, presenting ‘key uncertainties’ for discussion. Stakeholder discussions were facilitated (maximum of 8 per group) by a research team member using a broad topic guide (Additional file 1) based on the Normalisation Process Theory (NPT); a framework that utilises the core concepts of coherence, cognitive participation, collective action and reflexive monitoring to capture the work that participants do when implementing a new practice [34]. Verbal feedback and researcher field notes were collated and circulated to all stakeholders to ask for any further comment or clarification within a two-week deadline. Feedback was then mapped onto the NPT and reviewed against MoSCoW criteria for prioritising change [35], to support intervention refinement, as described above.

## Results

### Step one: understanding the behaviour

#### Identification, selection and specification of target behaviours

The results of the literature review and focus groups (reported elsewhere [9]) highlighted that most men on ADT are not receiving supervised exercise in line with NICE recommendation (NG131 1.4.19) [8]. Therefore, the target behaviour for men on ADT for prostate cancer was to attend twice weekly supervised exercise sessions, including aerobic and resistance training, for 12 weeks at Nuffield Health. The literature also identified reports of behaviours conducted by exercise professionals in supervising exercise. With the patient target behaviour in mind, these were distilled into 6 unique behaviours that CBEPs should perform. The final list of target behaviours were considered both measurable and modifiable and deemed most likely to bring about change (Table 1).

**Table 1:** Specification of target behaviours to focus upon in the STAMINA intervention

Target Behaviour	Behavioural specifications		
	Who	When	Where
1. To support patients first contact with the community gym providers	Fitness manager	On receipt of the patient referral	From the community-based gym, over the telephone
2. To conduct and record results of a fitness test	Exercise physiologist	At baseline and 12 weeks	At the community-based gym
3. To tailor the exercise prescription	Exercise physiologist	At baseline. Weekly reviews to determine tailoring requirements thereafter.	At the community-based gym
4. To deliver two supervised exercise sessions per week, per protocol	Personal trainer	Twice a week for 12 weeks	At the community-based gym
5. To provide weekly behavioural support	Exercise physiologist Personal trainer Fitness manager	At every contact	At the community-based gym and over the phone ad hoc
6. To compile progress reports and communicate progress with the clinical team	Exercise physiologist	At 12 weeks	At the community-based gym

#### Identifying what needs to change

Twenty-six men on ADT participated in one of five focus groups (age range 58–4 years, 96% White British, 36% no formal educational qualification, 64% retired) [9]. Thirty-four barriers to the patient target behaviour were identified, mapping onto 8 domains of the TDF, which highlighted domains requiring targeting (Additional file 2). These were: *knowledge, memory, attention and decision processes, beliefs about capabilities, beliefs about consequences, social/ professional role and identity, intention, reinforcement and emotion.*

Twenty-two Nuffield Health employed CBEPs participated in one of four focus groups in South Yorkshire, Derbyshire, North Somerset and the West of England (personal trainers *n* = 11, physiotherapists *n* = 4, physiologists *n* = 3, fitness managers *n* = 3 and general manager n = 1). Forty-one barriers to the CBEPs target behaviours were identified, that mapped onto 9 domains of the TDF, and thus highlighted domains with required targeting (Additional file 3). These were: *knowledge, skill, beliefs about capabilities, beliefs about consequences, social/ professional role and identity, intention, optimism, emotion and environmental context and resources.*

Furthermore, the CBEP focus groups revealed preference for the mode of delivery of the training package to include both face-to-face and online elements.

*“I’d much rather we come in and we all sit round and do it. I think with some phys-es like timewise it might be easier if they’ve got a bit of both. So, if you could have some online too”* (Personal Trainer)

#### Theoretical underpinning of the intervention

**Fig. 2:**
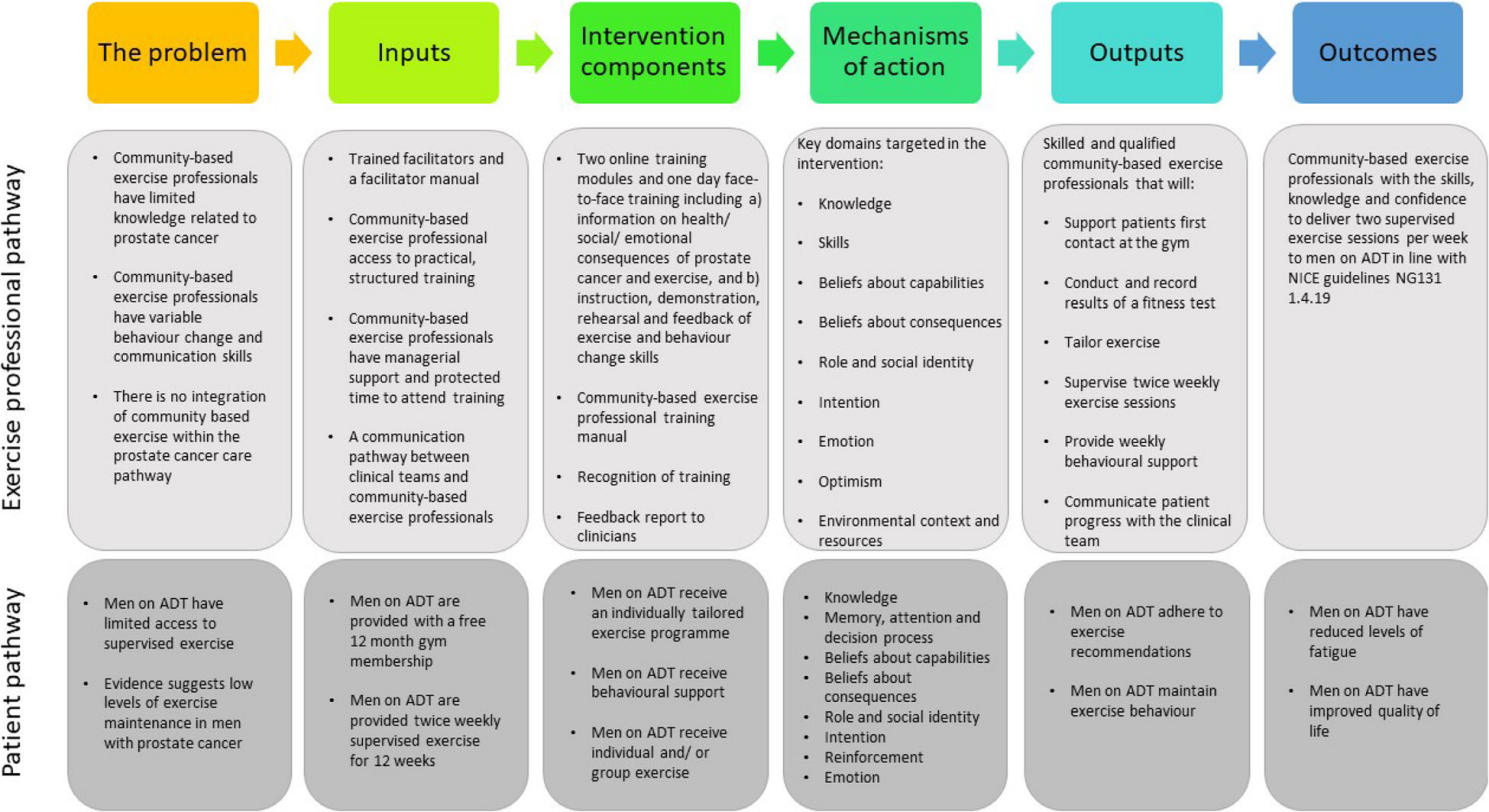
Logic model outlining the assumptions of a training package for community-based exercise professionals

Once the expert working group had identified key domains to target in the intervention, a logic model was developed outlining the assumptions of the intervention based on psychological theories (Fig. 2). The psychological theories that were explored in greater detail were: Social Cognitive theory (SCT: belief in capabilities and skill development) [36], Necessity Concerns Framework (NCF: belief in consequences and knowledge) [36] and Theory of Planned Behaviour (TPB: Professional role/ identity) [37]. Several constructs overlap between these theories e.g. perceived control (TPB) and self-efficacy (SCT), however there are also distinct constructs such as social norms (TPB), focus on skills training (SCT) and decisional balance (NCF) that suggested that whilst the models are complementary there was value in using each of these.

### Step two: identify behavioural content and implementation options

#### Behaviour change techniques

Twenty BCTs were identified to support delivery of the exercise professional training package. Due to the complexity of the intervention, constructs from more than one theory were used to underpin the behavioural content. For example, we included observational learning (i.e. modelling behaviour) with established techniques (i.e. demonstration of behaviour, verbal persuasion) to improve skills and beliefs about capabilities, based on Social Cognitive Theory [36]. To change beliefs about consequences of exercise, and improve knowledge, we provided information about health, social and emotional consequences in line with the Necessity and Concerns framework [38]. Furthermore, the Theory of Planned Behaviour suggests that behaviour is (indirectly) influenced by approval from important others in relation to whether a role should be performed (i.e. subjective norms) and thus we included credible sources to enhance motivation to adhere to the CBEP role [37].

Thirty-one BCTs were also identified to support behaviour change of men on ADT for prostate cancer (Additional file 4). For example, we included action planning, instruction on behaviour and self-reward due to their previous positive association with habit formation and exercise maintenance [39].

#### Mode of delivery

The exercise professional training package was developed to include 8 modules across 2 levels, in line with user preference and principles of blended learning. Level 1 was designed to be delivered individually, online, to provide flexibility for scheduling and completing tasks [40], and included introductory content. The more advanced content and integration of skill based practical learning formed level 2 which was designed to be delivered to groups (minimum 4, maximum 10), face-to-face, in a one-day interactive workshop. An overview of the behavioural content and mode of delivery of both levels is presented in Table 2.

**Table 2:** Overview of the exercise professional training package including summary of content and behaviour change techniques

Module (target behaviour)	Summary of content	BCTs (coded in line with BCTTV1)	TDF domain
**Level 1: online**			
Module 1: Introduction to prostate cancer and exercise (1)	- Provide an overview of the STAMINA programme and NICE guidelines (text and video from consultant urologist) - Provide evidence-base for exercise as a treatment component (text and video from consultant urologist) - Present patient views on exercise (quotes) - Present information about prostate cancer, symptoms, mortality, treatment and side effects (text and video from consultant urologist) - Present information on the role of CBEPs in STAMINA (video from consultant urologist) - Summary of key points	5.1 Health consequences, 5.3 Social & environmental consequences	Knowledge
		4.1 Instruction, 9.1 Credible source	Professional role
		5.6 Emotional consequences, 9.1 Credible source, 10.1 Material incentive	Beliefs about consequences
Module 2: Operationalising the STAMINA programme (1, 4)	- Present information on communication with clinical teams (text and video from consultant urologist) - Present information on scheduling STAMINA patients for exercise - Present frequently asked questions and answers related to patient memberships, appointments and missed sessions.	5.1 Health consequences, 5.3 Social & environmental consequences	Knowledge
		4.1 Instruction	Skill
		4.1 Instruction, 9.1 Credible source	Professional role
		5.6 Emotional consequences, 9.1 Credible source	Beliefs about consequences
**Level 2: Face-to-face**			
Module 3: Working with clinical populations (1)	- Discussion on prostate cancer treatment, side effects, symptoms, survival rates and role of exercise. - Discussion about patient feelings towards attending the gym and challenge beliefs - Present detailed information about side effects (case studies) - Discuss how best to respond to different side effects and consider pros and cons of each - Provide instruction and demonstration on active listening and helpful phrases to use	2.2 Feedback on behaviour, 5.1 Health consequences, 5.3 Social & environmental consequences	Knowledge
		4.1 Instruction, 6.1 Demonstration	Skill
		4.1 Instruction, 9.1 Credible source	Professional role
		9.1 Credible source	Intention
		5.6 Emotional consequences, 9.1 Credible source, 9.2 Pros and cons, 10.1 Material incentive	Beliefs about consequences
		5.6 Emotional consequences, 9.1 Credible source, 11.2 Reduce negative emotions	Emotion
		3.2 Social support, 12.5 Adding objects	Environmental context
Module 4: Tailoring the exercise prescription (3)	- Instruction on professional role - Discussion related to previous similar experiences and persuasion about capability - Demonstrate safe tailoring (case study) - Beliefs about patient capabilities discussed and challenged (case studies) - Evidence based information about tailoring for clinical populations provided in a handbook and pros and cons discussed - Information and example of a run-in period provided and discussed - Practical task with feedback - Access to case report forms and instruction on how to access NHS.net	2.2 Feedback on behaviour, 4.1 Instruction, 5.1 Health consequences, 5.3 Social & environmental consequences, 5.6 Emotional consequences	Knowledge
		2.2 Feedback on behaviour, 4.1 Instruction, 6.1 Demonstration, 8.1 Rehearsal	Skill
		4.1 Instruction, 15.1 Verbal persuasion	Professional role
		9.1 Credible source, 9.2 Pros and cons	Beliefs about consequences
		3.3 Social support, 11.2 Reduce negative emotions	Emotion
		12.5 Adding objects	Environmental context
Module 5: Delivering the exercise prescription (4)	- Discussion about consequences of monitoring exercise and previous experience, focussing on success - Discussion about challenges of delivering to small groups and persuasion of capability - Instruction and demonstration of recording heart rate and exertion - Practical graded task delivering and recording tailored exercise to small groups, with feedback - Information that protected time to deliver exercise will be provided at an increased salary	2.2 Feedback on behaviour, 4.1 Instruction, 8.1 Rehearsal, 8.7 Graded task	Skill
		2.2 Feedback on behaviour, 4.1 Instruction, 8.1 Rehearsal, 8.7 Graded task, 15.1 Verbal persuasion, 15.3 Focus on past success	Beliefs about capabilities
		5.1 Health consequences, 5.3 Social & environmental consequences, 5.6 Emotional consequences, 9.1 Credible source, 10.1 Material incentive, 10.8 Incentive	Beliefs about consequences
		3.3 Social support, 11.2 Reduce negative emotions	Emotion
		3.2 Social support, 12.1 Restructure, 12.5 Adding objects	Environmental context
Module 6: Reviewing the exercise prescription (6)	- Discuss importance of reviewing exercise - Provide instruction on professional role - Provide instruction and demonstration of completing a progress report (case study) - Role play task: completing progress reports and discussing progress. Feedback provided. - Discuss importance of language used - Verbal persuasion about capabilities and focus on past success	2.2 Feedback on behaviour, 4.1 Instruction, 6.1 Demonstration, 8.1 Rehearsal	Skill
		4.1 Instruction	Professional role
		15.1 Verbal persuasion	Optimism
		3.3 Social support, 11.2 Reduce negative emotions, 15.1 Verbal persuasion	Emotion
		12.5 Adding objects	Environmental context
Module 7: Behaviour change (5)	- Discuss what behaviour is, how it is influenced and how it can be changed, focus on past success - Introduce behaviour change theory - Discuss capability, opportunity and motivation of men on ADT and challenge beliefs (case study videos) - Present patient video discussing the importance of the exercise professional role and behavioural support - Role play task: respond to exercise ambivalence/ resistance (case studies). - Review task in group discussion and provide feedback. - Provide instruction/ demonstration of suitable BCTs for exercise ambivalence and resistance - Role play task: repeated with new skills learnt - Discussion and video examples of good and bad communication focusing on the spirit of motivational interviewing	2.2 Feedback on behaviour, 4.1 Instruction, 5.6 Emotional consequences	Knowledge
		1.2 Problem solving, 2.2 Feedback on behaviour, 4.1 Instruction, 6.1 Demonstration, 8.1 Rehearsal, 8.7 Graded task	Skill
		4.1 Instruction	Professional role
		2.2 Feedback on behaviour, 4.1 Instruction, 6.1 Demonstration, 8.1 Rehearsal, 8.7 Graded task, 15.1 Verbal persuasion, 15.3 Focus on past success	Beliefs about capabilities
		5.1 Health consequences, 5.6 Emotional consequences, 9.1 Credible source	Beliefs about consequences
Module 7: Fitness Testing (2)	- Introduce the fitness test and provide handouts (protocol, exertion scale, case report form) - Provide instruction and demonstration on the gym floor - Discuss safe parameters of exercise testing (case study) - Access to gym floor and heart rate monitors for practical, graded task with feedback using case report forms to record outcomes (i.e. heart rate, exertion, time, level)	2.2 Feedback on behaviour, 4.1 Instruction, 5.1 Health consequences	Knowledge
		2.2 Feedback on behaviour, 4.1 Instruction, 6.1 Demonstration, 8.1 Rehearsal, 8.7 Graded task	Skill
		4.1 Instruction	Professional role
		5.6 Emotional consequences, 9.1 Credible source	Beliefs about consequences
		11.2 Reduce negative emotions	Emotion
		12.1 Restructure environment, 12.5 Adding objects	Environmental context

The BCTs included to support patients exercise behaviour were designed to be delivered face-to-face by CBEPs or presented interactively in a patient journey booklet designed as a behaviour change and self-monitoring tool (Additional file 4).

### Step 3: delivery and refinement of the intervention

The intervention was refined following feedback from CBEPs and stakeholders. A summary of the main changes is provided below, with further detail in Additional file 5.

#### Rehearsal delivery

Eleven CBEPs attended the rehearsal training and subsequent focus group. Feedback was categorised into three themes: 1) *Content* - CBEPs requested contact details to signpost men for further support outside of the exercise professional role (e.g. bone pain or change in medication); 2) *Format* - CBEPs requested additional time to complete interactive tasks, with a focus on behavioural rehearsal; 3) *Recognition* - CBEPs enjoyed the training programme but requested accreditation for completion of training as an incentive/ recognition for participation and to enhance trust in the referral pathway. All suggestions were deemed high priority and repeated by several participants thus strategies to implement these changes were made such as providing additional time to facilitate learning and providing CBEPs with a title change on completion of training (i.e. clinical exercise specialist).

#### Stakeholder workshop

Feedback from 28 stakeholders (i.e. HCPs, academics, researchers, PPI members, members of local charities, commissioners, senior community-gym staff and CBEPs) was mapped onto the four domains of the NPT (S7): 1) *Coherence –* it is important to include the evidence-base of STAMINA; 2) *Cognitive participation* – CBEPs should receive recognition for training completion 3) *Collective action -* personal trainers possess the skills to fulfil the role of the physiologist and have more time to deliver the intervention; 4) *Reflexive monitoring* – Level 1 training should be uploaded onto internal systems for ease of access. The feedback, repeated by several participants, mirrored suggestions from CBEPs and were deemed high priority. In response, we provided personal trainers the opportunity to apply for the role of the physiologist, creating an application form with essential and desirable criteria to ensure sufficient skill set ahead of further professional training.

The final intervention is presented in Additional file 6 in line with the Template for Intervention Description and Replication (TIDieR) [41].

## Discussion

This paper describes the methods used and outputs realised from the development and refinement of a theory and evidence-based training package for CBEPs to support the delivery of NICE recommendations (NG131 1.4.19), prior to full effectiveness and implementation evaluation in a planned randomised controlled trial. We demonstrate a systematic approach to intervention development incorporating stakeholder, PPI and user perspectives to optimise safe delivery of a disease specific, tailored exercise programme. Both the process and intervention, if shown to be effective, have the potential to be adapted for exercise interventions with other clinical populations.

To date little is known about the contextual factors that may influence implementation of exercise into the prostate cancer care pathway, in partnership with community gym providers, due to its novelty. In this paper we address this uncertainty by adopting a user-centred design, collecting in-depth qualitative research iteratively, (guided by behavioural theory) to understand user views, context, and experience [19]. This method has similarities to Chaudoir and colleagues’ multilevel framework [42], whereby we collected qualitative data from three levels hypothesised to influence implementation outcomes: at the patient, provider and organisational (albeit limited) level [42–44]. Subsequently, we developed an in-depth understanding of patient and professional needs to specify key modifiable behaviours and intervention content that was deemed acceptable, to support behaviour change. More specifically, we designed a training package for CBEPs to address previously reported gaps by incorporating the core competencies of working with clinical populations [16]. A blended learning approach was followed to accommodate different learning styles, and provide opportunity for real-time reflection, questions and discussion [45]. This approach may be advantageous in the current climate whereby social distancing measures are common and digital health/ interventions are rapidly being adopted due to Covid-19 [46]. However, further refinements to the intervention may be required if remote delivery extends to clinical exercise services for people with cancer.

Feedback from intervention users and stakeholders highlighted modifications which could further improve intervention effectiveness and implementation into the real-world. For example, integration of an exercise referral pathway between two currently distinct sectors (i.e. the NHS and leisure industry) may benefit from formal recognition/ accreditation of professional training to reduce previously reported concerns regarding CBEPs skills to deliver a safe, and disease specific exercise programme [9]. Furthermore, uploading online training content to the university as opposed to the organisation’s website was perceived as a potential barrier, which if addressed, could further optimise implementation. These observations will require confirmation in our planned definitive randomised controlled trial whereby treatment fidelity will be measured, i.e. professional’s application and delivery of the skills learnt in training into real-life context [47] and a qualitative process evaluation conducted to identify areas that may require further refinement [27].

### Implications for implementation and future work

To date, exercise is the only evidence-based treatment to help manage side effects of ADT [6, 7]. However, exercise is rarely offered as standard treatment for prostate cancer [9] and many barriers to implementation exist when delivered by exercise professionals working in health services, including: pathway inadequacy, limited scope for scalability and limited skillset, i.e. delivered to a more limited range of clinical populations than efficacy studies and clinical guidelines indicate would be beneficial. We demonstrate a potential solution – the development of a referral pathway between two currently distinct sectors, which, when combined with professional training (HCP and CBEP) as part of a complex intervention, can support the delivery of NICE recommendations nationally. The next steps are to test the effectiveness and cost-effectiveness of the intervention in a definitive randomised trial. We will also conduct a process evaluation focussing on implementation outcomes such as how well the intervention is adopted in services and if it is seen as acceptable and appropriate by stakeholders, CBEPs and patients. In this sense the trial can be considered to adhere to a type 1 hybrid design [48] which is increasingly recommended for trials of this sort.

We also emphasise the importance of combining formalised intervention development approaches to ensure the approach is systematic, user centred, theory and evidence based and conducted at multilevel to enhance implementation. This approach allows for interventions to be refined and adapted to reach larger target audiences, i.e. to support other clinical populations where exercise is beneficial but under-utilised.

### Strengths and limitations

The methods reported in this paper were based on a combination of formalised intervention development approaches [18, 19], to address limitations of each approach. For example, the BCW provides a systematic process to intervention development and is presented as a simple stepwise process to enhance usability. However, optimal intervention development takes a much more lengthy, non-linear process [49] and thus we took an iterative approach to intervention development as suggested by the MRC guidance for the development of complex interventions [17]. We also included components of the PBA to gain ongoing feedback from user perspectives to optimise future implementation. Furthermore, we addressed previously reported barriers to integrating NICE recommendations into practice [10, 11], by developing an intervention to be delivered by community providers; to enhance patient accessibility and scalability during the implementation process [50]. Furthermore, NHS services are currently under extreme pressure due to the coronavirus pandemic [51] thus highlighting the need to explore new pathways of care.

A common limitation of intervention development approaches is the limited guidance, or evidence to support the selection of BCTs in clusters, and the unclear instructions on how to select and apply appropriate theories to support intervention content. For example, most theories predict behaviour as opposed to providing clear instructions and techniques on how to change behaviour [52]. However, the Social Cognitive Theory [36] is an exception as it provides examples of techniques to influence behaviour change. To further improve the generalisability of our methods we took a triangulation approach reviewing evidence-based links [29] and most frequently used BCTs [18] against pre-defined criteria (i.e. APEASE) and ensured consensus was achieved amongst our expert working group.

## Conclusion

This paper describes the development and refinement of a training package to support CBEPs implementing NICE recommendations into prostate cancer care. We demonstrate how established intervention development approaches can be combined to provide a systematic approach to developing a theory and evidence-based intervention and emphasise the importance of incorporating stakeholder, PPI and user perspective to ensure the intervention remains relevant and acceptable to the recipient during each refinement iteration. Both the process and intervention have the potential to be adapted for exercise interventions with other clinical populations where exercise is recommended but under-utilised.

## Supplementary Information

**Additional file 1.** Topic guides. This file contains three topic guides that were used for primary qualitative data collection in the intervention development and refinement process 1) Focus groups pre-rehearsal delivery to community-based exercise professionals, 2) Focus groups post-rehearsal delivery to community-based exercise professionals and 3) Round table discussion during the stakeholder workshop.

**Additional file 2.** Barriers to exercising twice weekly as reported by men on androgen deprivation therapy for prostate cancer: mapped onto the Theoretical Domains Framework. This file contains a list of barriers and example quotes to exercising twice weekly in a gym, reported by men on androgen deprivation therapy for prostate cancer. Responses are mapped onto the Theoretical Domains Framework.

**Additional file 3.** Barriers to supervising exercising twice weekly as reported by community-based exercise professionals: mapped onto the Theoretical Domains Framework. This file contains a list of barriers and example quotes to supervising men on androgen deprivation therapy for prostate cancer to exercise twice weekly in a gym, reported by community-based exercise professionals. Responses are mapped onto the Theoretical Domains Framework.

**Additional file 4.** Summary of the patient intervention. This file provides a summary of the behavioural content of the patient intervention.

**Additional file 5.** Intervention refinements based on feedback from community-based exercise professionals and stakeholders. This file provides feedback on the intervention (training package) following rehearsal delivery to community-based exercise professionals and presentation to stakeholders. Feedback is collated into key themes. Where modifications were made, criteria for change and a description of the impact upon the intervention is provided.

**Additional file 6.** Template for Intervention Description and Replication (TIDieR) of the community-based exercise professional intervention. This file provides an overview of the community-based exercise professional intervention (training package).

## Data Availability

The datasets supporting the conclusions of this article are included within the article (and its additional files).

## Abbreviations

APEASE: Affordability, Practicability, Effectiveness and Cost-effectiveness, Acceptability, Side effects and Safety, Equity considerations
ADT: Androgen Deprivation Therapy
BCTs: Behaviour Change Techniques
BCW: Behaviour Change Wheel
CBEP: Community-based exercise professional
HCP: Healthcare professional
MoSCoW: Must have, Should have, Could have, Would like
MRC: Medical Research Council
NICE: The National Institute for Health and Care Excellence
NPT: Normalisation Process Theory
PPI: Patient and Public Involvement
QoL: Quality of Life
STAMINA: Supported exercise TrAining for Men wIth prostate caNcer on Androgen deprivation therapy
TDF: Theoretical Domains Framework
TIDieR: Template for Intervention Description and Replication
UK: United Kingdom
