# Supplementary material for: Towards implementing exercise into the prostate cancer care pathway: development of a theory and evidence-based intervention to train community-based exercise professionals to support change in patient exercise behaviour (The STAMINA trial)
Source: BMC Health Serv Res. 2021 Mar 22;21:264. doi: 10.1186/s12913-021-06275-w (PMC7982309; doi:10.1186/s12913-021-06275-w)
Supplement: Supplementary file 1 — Additional file 1. Topic guides. This file contains three topic guides that were used for primary qualitative data collection in the intervention development and refinement process 1) Focus groups pre-rehearsal delivery to community-based exercise professionals, 2) Focus groups post-rehearsal delivery to community-based exercise professionals and 3) Round table discussion during the stakeholder workshop. [file 12913_2021_6275_MOESM1_ESM.docx]

**Additional file 1: Topic guides**

File 1a: Focus group topic guide for exercise professionals (pre-training) based on the Theoretical Domains Framework (1)

*A brief overview of the study will be given to the participants followed by general house-keeping rules and focus group etiquette. There will be an opportunity for the participants to ask questions before starting. They will also be reminded that the focus group will be audio recorded.*

**Job role as an exercise professional and working with men with PCa**

- If you could please tell us about your current job roles?
- Can you tell me about the sort of clients you usually work with?
  - PROBE: elderly, with health problems, cancer (Social/ professional role and identity)
  - PROBE: can you give some examples?
- Are there things you have to take into account with these populations
  - PROBE: practical, psychological, other barriers
- How motivated do you find your clients from a clinical population? How do you address this (Skill)
  - PROBE: what have you found to be successful and not successful? Are there any common barriers you would anticipate?
  - PROBE: specific behaviour change techniques
- What things do you think can help this population to exercise?
- PROBE: giving rewards/incentives to individuals taking part in exercise? (Reinforcement)
- How would you tailor an exercise prescription to meet an individual’s needs? (Skill)
- PROBE: to cancer specific needs?
- What are your thoughts on someone exercising when they have cancer (Beliefs about consequences)
- PROBE: Do you know about the common adverse effects a man with prostate cancer on hormone therapy may suffer with?
- PROBE: What effect do you think exercise could have on this patient group? (Beliefs about consequences)
- Would working with men with advanced prostate cancer evoke an emotional response in you? (Emotion)
- How would you feel if some of the men shared worries and concerns about their condition with you? (Emotion)
  - PROBE: How confident are you (have skills to manage) in this?
- What areas of advice would you see as part of your role as an exercise professional?
  - PROBE: Nutrition, sleep, stress management – give examples of sort of things you do
- What would you see as your role for this patient group? Would you want to provide advice other than exercise? (Social influence)

**Clinical team communication**

- When you’ve worked with patients with clinical problems what interaction do you normally have with the clinical team
  - What works/doesn’t/ideas for success. Have you previously worked alongside a clinical team to deliver exercise?

PROBE: what went well and what didn’t go so well?

- How have you previously communicated with health care professionals about a patient on a research project before? (Memory, attention and decision process)
- Would you feel confident in working alongside a clinical team? (Beliefs about capabilities)
- Would you anticipate any barriers? (Environmental context and resources) How could these be overcome?
- Do you think there would be any barriers from health care professionals? (Environmental context and resources)
- If you were designing a regular reporting system to form an embedded link between NH teams and NHS PCa teams, how would you design it in an ideal world?

**Research experience**

- Have you previously worked on research projects?
- Do you usually work to research protocols? (Memory, attention and decision processes) Are you trained in Good Clinical Practice? (Memory, attention and decision processes)
- What do you know about research protocols? (Knowledge)
- What conflicts can you see arising with your normal working practices and working to a pre-defined research protocol?

PROBE: Is there anything you could do or would need to improve your confidence? (Beliefs about capabilities)

**Overview**

Would you be interested in being involved in such a programme? What would be your main motivations for this? (Goals)

- Would you be happy to take on further training? Would there be any specific areas you would feel you need to improve before working on this programme? (Skill)

PROBE: How would this best be delivered? Face to face or online?

- Is there anything else you like to add or discuss?

File 1b: Focus group topic guide for exercise professionals (post-training) based on Kirkpatrick (2)

*A brief overview of the study will be given to the participants followed by general house-keeping rules and focus group etiquette. There will be an opportunity for the participants to ask questions before starting. They will also be reminded that the focus group will be audio recorded.*

- How do you feel about the training experience?
  - Content - Was it understandable? Was it enjoyable?
  - Delivery - Was it pitched at the correct level?
  - Was the duration correct? Delivered by right personnel
- Did participants feel they had learned something new from the training? What new skills/information do you feel you have learnt from the course?
- probe (different skills targeted)
- Do you think there are other topics/skills that would have been useful to include in the training?
- Were there any aspects of the course which you didn’t find helpful?
- How confident do you feel in being able to use these skills as part of the STAMINA intervention/ in routine practice
  - What would increase your confidence?
- How motivated do you feel to deliver STAMINA within your team?
- Probe, all members of team
- Anything that could aid motivation
- Do you see any barriers to using these skills in practice/ Are there any things which could make it easier?
  - How do you feel patients will respond to this new approach?
  - Helpful?
  - Acceptable to patients?
- Would you recommend the course to others?

File 1c: Focus group topic guide for the stakeholder workshop based on the Normalisation Process Theory (3)

*A brief overview of the study will be given to the participants followed by general house-keeping rules and focus group etiquette. There will be an opportunity for the participants to ask questions before starting.*

- Opinions on the proposed content, format and structure of the intervention (HCP, patient and exercise professional elements and communication pathways)
- Does the intervention have a clear purpose for all participants (HCPs in the MDT, exercise professional and study patient participants)? (*COHERENCE*)
- How feasible is the delivery of the intervention (*COGNITIVE PARTICIPATION*, *COLLECTIVE ACTION*)
  - Opinions on the mode of delivery of intervention?
  - Opinions on the duration of intervention?
- Do participants believe the intervention will be put in place (*COGNITIVE PARTICIPATION, COLLECTIVE ACTION*)
  - What would be peoples’ motivations, barriers, capabilities to put in place?
  - Does intervention fit with individuals’ roles? Does the intervention fit with the overall organizational goals of the hospital team and the exercise professionals?
- Do participants believe the intervention will bring benefits (and be perceived as advantageous) for patients, for staff and for organisations? (*COHERENCE, REFLEXIVE MONITORING*)
  - How will benefits be recognised,
  - Ways to facilitate this

- How acceptable is the new HCP behaviour likely to be to patients (the ultimate recipients)? (*COGNITIVE PARTICIPATION*)
- How acceptable is the communication pathway likely to be to patients? (*COLLECTIVE ACTION*)

- Opinions on the proposed pathways of communication between health care professional and exercise professionals (*COLLECTIVE ACTION*)
  - What are the best ways of communicating between these two groups?
  - What is feasible?
  - How acceptable is this communication, to patients?
- How might the fidelity of the intervention be promoted? (*COLLECTIVE ACTION*)
- Will it be clear from the study what effects the intervention has had, will the team be aware of benefits? (*REFLEXIVE MONITORING*)
- Is there learning from related areas that could be helpful here e.g. cardiac rehabilitation. (*REFLEXIVE MONITORING*)

**References**

1. Michie S, Johnston M, Abraham C, Lawton R, Parker D, Walker A. Making psychological theory useful for implementing evidence based practice: A consensus approach. In: Quality and Safety in Health Care. BMJ Publishing Group Ltd; 2005. p. 26–33.

2. Kirkpatrick DL. Evaluating training programs: evidence vs. proof. Train Dev J [Internet]. 1977 [cited 2020 Oct 9];9–12. Available from: https://agris.fao.org/agris-search/search.do?recordID=US201302470542

3. Murray E, Treweek S, Pope C, MacFarlane A, Ballini L, Dowrick C, et al. Normalisation process theory: A framework for developing, evaluating and implementing complex interventions. BMC Med [Internet]. 2010 Oct 20 [cited 2020 Jul 7];8(1):1–11. Available from: https://link.springer.com/articles/10.1186/1741-7015-8-63
