# Supplementary material for: Towards implementing exercise into the prostate cancer care pathway: development of a theory and evidence-based intervention to train community-based exercise professionals to support change in patient exercise behaviour (The STAMINA trial)
Source: BMC Health Serv Res. 2021 Mar 22;21:264. doi: 10.1186/s12913-021-06275-w (PMC7982309; doi:10.1186/s12913-021-06275-w)
Supplement: Supplementary file 2 — Additional file 2. Barriers to exercising twice weekly as reported by men on androgen deprivation therapy for prostate cancer: mapped onto the Theoretical Domains Framework. This file contains a list of barriers and example quotes to exercising twice weekly in a gym, reported by men on androgen deprivation therapy for prostate cancer. Responses are mapped onto the Theoretical Domains Framework. [file 12913_2021_6275_MOESM2_ESM.docx]

**Additional file 2: Barriers to exercising twice weekly as reported by men on androgen deprivation therapy for prostate cancer: mapped onto the Theoretical Domains Framework**

| **TDF Domain** | **Barriers** | **Example Quotes** |
| --- | --- | --- |
| Knowledge | 1. Uncertainty of ADT side effects 2. Lack knowledge of exercise recommendations 3. Uncertain of what constitutes moderate intensity exercise 4. Uncertainty related to exercise as a treatment component for PCa | *“I’ve had prostate cancer for about a year but I didn’t really know whether exercise would help it”*  *“Is there any exercise that’s particularly associated or helps with prostate cancer?”* |
| Memory, attention and decision process | 1. Avoid talking about PCa 2. Avoid thinking about PCa 3. Avoid information related to PCa, including treatment 4. Lack ability to discern between ADT side effects, injury and cancer reoccurrence | *“Well I just don’t want to talk about it. I don’t want to acknowledge that I’ve got it”* |
| Beliefs about capabilities | 1. Not capable of exercising for long durations 2. Not capable of lifting heavy weights 3. Not capable of exercising in a gym | *“I used to be a lorry driver and I used to do the same, lifting heaving metal bars and I couldn’t do anything like that now”* |
| Intention | 1. No intention to exercise as a treatment component | *“I don’t want to commit to your trial, because the next year is so important for me that I’d just rather do my own thing”* |
| Reinforcement | 1. Men are more likely to exercise if the programme is individually tailored to them 2. Men are les likely to maintain exercise behaviour if progress is not demonstrated/ felt. | *“The key thing that would make me engage with it, is if it was individualised to me”*  *“Every time I take some physical stuff up I am looking for something to advance me really and nothing is happening”*  *“You think the main incentive would then be feeling better in yourself and also seeing some improvements”* |
| Beliefs about consequences | 1. Healthcare professionals will not advocate exercise 2. Belief that some men would prefer to use their spare time doing alternative things (e.g. holiday) 3. Belief that a gym membership may be too expensive 4. Belief that side effects of treatment may reduce ability to exercise 5. Lack clear understanding of the benefits of exercise (general) 6. Lack understanding of the benefits of exercise for men on ADT (specific) | *“My doctor who is not the fittest person and his attitude to fitness is, “Don’t burn yourself out. You have still got a long way to go, don’t think you can do it.” He is more off-putting than me doing it”*  *“For me having to pay is no problem whatsoever, but I can imagine people who might find having to pay normal gym fees a financial strain”* |
| Social identity | 1. Self-identify as sedentary 2. Self-identify as reduced functional capacity 3. Self-identify as unfit 4. Do not self-identify as a member of a gym | *“I wouldn’t say I’m generally fit. I wouldn’t say that at all”*  *“Saying with gym work I have done all the activities that have kept relatively fit, but I have never really been a gym person”* |
| Emotion | 1. Fear of over-exertion 2. Worry of pain in the groin area 3. Embarrassed of feeling emotional 4. Lack motivation to get out of bed (some days) | *“I can find myself in tears over that to see how we’ve deteriorated in health. But it is something that comes on me very quickly. Which I find embarrassing as well, but I can’t control it”* |
