# Supplementary material for: Towards implementing exercise into the prostate cancer care pathway: development of a theory and evidence-based intervention to train community-based exercise professionals to support change in patient exercise behaviour (The STAMINA trial)
Source: BMC Health Serv Res. 2021 Mar 22;21:264. doi: 10.1186/s12913-021-06275-w (PMC7982309; doi:10.1186/s12913-021-06275-w)
Supplement: Supplementary file 3 — Additional file 3. Barriers to supervising exercising twice weekly as reported by community-based exercise professionals: mapped onto the Theoretical Domains Framework. This file contains a list of barriers and example quotes to supervising men on androgen deprivation therapy for prostate cancer to exercise twice weekly in a gym, reported by community-based exercise professionals. Responses are mapped onto the Theoretical Domains Framework. [file 12913_2021_6275_MOESM3_ESM.docx]

**Additional file 3:** **Barriers to supervising exercising twice weekly as reported by community-based exercise professionals: mapped onto the Theoretical Domains Framework**

| **Theoretical Domains** | **Barriers** | **Example Quotes** |
| --- | --- | --- |
| Skills | 1. Lack skills delivering behaviour change techniques 2. Possible lack of skill conducting submaximal bike and treadmill tests 3. Lack of experience communicating progress to clinical teams | *“Not fitness testing though, we don’t have, probably don’t have quite the knowledge base or the facilities to do that”* (Physiotherapist) |
| Knowledge | 1. Lack knowledge related to PCa treatment, side effects and mortality 2. Lack understanding of safe parameters of exercise testing for men on ADT 3. Lack understanding of safe parameters of exercise training for men on ADT 4. Lack knowledge of the evidence base of exercise for men on ADT 5. Uncertainty of men on ADT physical capabilities’ 6. Perceived lack of knowledge related to communicating about PCa and exercise 7. Perceived lack of specialist clinical knowledge 8. Not recognising how or when to use BCTs to support individuals ambivalent to change | *“I think as well like I think there’s a tiny gap in my knowledge for what physiologically might happen to somebody having treatment for prostate cancer”* (Personal Trainer)  *“I have no idea off the top of my head. I would assume maybe sitting on a bike’s probably uncomfortable, so you’d probably want to go away from that, but that’s dealing with a layman’s logical fallacy. It might not be true at all, but I have no real clue”* (Personal Trainer)  *“Well, I guess if we were educated, we would know the survival rates and things like that”* (Personal Trainer) |
| Social identity and professional role | 1. Physiologists believe PTs can fulfil their role 2. Physiologists believe PTs lack time to provide emotional/ behavioural support on the gym floor 3. Exercise professionals perceive their role to include elements of counselling 4. Exercise professionals identify men on ADT to be motivated to exercise | *“What would the physiologist be adding that the PT can’t do”* (Physiologist)  *“I know cancer can have a large toll on people’s wellbeing, on people’s psyche. So the person who is training them, they’re going to be like a one-to-one person, almost like a counsellor or like a psychiatrist”* (Personal trainer) |
| Beliefs about capabilities | 1. Belief that men on ADT have limited physical capabilities 2. Lack of confidence using BCTs 3. Lack of confidence supervising exercise for men on treatment for cancer | *“I’d say you’d expect someone, depending on what management they’d had for cancer, they’re going to be really deconditioned”* (Physiotherapist)  *“So, our qualifications are quite broad. So, I think for all of us to feel confident we’d need quite in-depth training on that specific cancer”* (Personal trainer) |
| Optimism | 1. Perception that communicating with clinical teams will be challenging | *“We still see challenges in bridging that gap between medical and wellbeing, if you like, and that’s in an already integrated company, so I could see that that’s going to be the challenge”* (General Manager) |
| Intention | 1. Belief that men on ADT will not want to exercise in a gym | *“What was going to be my idea is that I know that people are going to be referred, but would they all actually go”* (Personal Trainer) |
| Beliefs about consequences | 1. Belief that handovers between exercise professionals will be time consuming 2. Belief that scheduling appointments with the physiologist will be challenging 3. Belief that receiving referrals during peak times will be challenging 4. Beliefs that exercise professionals will want to participate for an increased salary only 5. Belief that exercise professionals will not participate if there is no incentive i.e. change in title 6. Belief that PTs do not have time to deliver supervised exercise on shift 7. Belief that PTs will not deliver optimal care unless paid in line with a PT salary 8. Belief that anxieties of men on ADT will negatively impact the EPs 9. Belief that men on ADT will feel uncomfortable if seen to be treated differently on the gym floor 10. Belief that men on ADT will stop exercising if goals are not met 11. Belief that men will not be open about their thoughts and feelings 12. Belief that men on ADT will be happier where there is continuity in care | *“I don’t think it would work being on shift. There are just not enough hours in a shift to do this”* (Personal Trainer)  *“I’ve directly dealt with people who have had prostate cancer in the past… And those anxieties are then pushed on to us, because then you go ooh I don’t know if I’m pushing that person too hard, could that weaken them further”* (Personal Trainer)  *“Basically, the other guys will say we get paid a bit more for doing PT sessions than when we’re on shift. So, if we’re on shift having to do two extra PT sessions a week, they’ll want the same pay as a PT session basically”* (Personal Trainer)  “*So, you know, if somebody is being seen as being treated differently on the gym floor in front of other people, especially if you require further equipment, so I think some people won’t worry too much about that, and then other people will have a self-confidence issue with that, I guess”* (Physiologist) |
| Emotion | 1. Exercise professionals are nervous about working alongside clinical teams 2. Exercise professionals are nervous about doing something wrong and having a negative impact 3. Belief that men on ADT will be anxious about exercising 4. Belief that men on ADT will be anxious about being in a gym environment | *“The last thing you want to do is something detrimental to that person unknowingly, so yeah it is quite scary. It’s similar working with pregnant people. It’s just God forbid you did something that would hurt somebody, so there is definitely an underlying anxiety”* (Personal Trainer)  *“I think it’s more of a psychosocial issue and getting over the fear and anxiety of being in a new environment”* (Physiotherapist) |
| Environmental context and resource | 1. Limited access/ availability to personal emotional support 2. Limited community gyms have on site physiologists 3. High staff turnover in some community gyms 4. No access to secure, encrypted email for communication with the NHS | *“I don’t think there’s any support for us at all, that I’m aware of if things go a bit awry at all, so I think that’s really important”* (Personal Trainer)  *“I think logistics also has to be the key issue as well, because for example us, like we don’t have a physiologist”* (Personal Trainer) |
