# Supplementary material for: Towards implementing exercise into the prostate cancer care pathway: development of a theory and evidence-based intervention to train community-based exercise professionals to support change in patient exercise behaviour (The STAMINA trial)
Source: BMC Health Serv Res. 2021 Mar 22;21:264. doi: 10.1186/s12913-021-06275-w (PMC7982309; doi:10.1186/s12913-021-06275-w)
Supplement: Supplementary file 4 — Additional file 4. Summary of the patient intervention. This file provides a summary of the behavioural content of the patient intervention. [file 12913_2021_6275_MOESM4_ESM.docx]

**Additional file 4: Summary of the patient intervention**

| **Overview of patient intervention** | **Mode of delivery** | **BCTs (coded in line with BCTTV1)** |
| --- | --- | --- |
| 1. Patient is contacted by the fitness manager to check expectations, schedule first appointment and prompt use of interactive booklet. | Over the telephone with exercise professional | 1.2 Problem solving, 1.4 Action planning, 3.3 Social support, 5.1 Health consequences, 6.3 Others approval |
| 1. Patient attends an induction session at the gym which includes the following:  - Discuss patient attitude and expectations - Explain membership and access - Explain sign in process - Tour/ Induction to gym and facilities - Submaximal test - Tailor the exercise prescription - Discuss session timetable and choose sessions - Schedule patient review | Face-to-face with exercise professional on the gym floor | 1.2 Problem solving, 1.4 Action planning, 1.9 Commitment, 2.1 Monitoring behaviour by others, 2.2 Feedback, 4.1 Instruction, 5.1 Health consequences, 5.3 Social & environmental consequences, 5.6 Emotional consequences, 6.1 Demonstration, 6.3 Others approval, 8.1 Rehearsal, 8.7 Graded task, 9.2 Pros and cons, 12.5 Adding objects, 15.1 Verbal persuasion |
| 1. Patient attends twice weekly supervised exercise sessions combining aerobic and resistance training for 12 weeks. Each session lasts approximately 1 hour. | Face-to-face with exercise professional on the gym floor, either one-to-one or in small groups with other patients in the study. | 2.1 Monitoring behaviour by others, 2.2 Feedback, 2.6 Biofeedback, 2.7 Feedback on outcome, 3.3 Social support, 4.1 Instruction, 6.1 Demonstration, 8.1 Rehearsal, 8.3 Habit formation, 15.1 Verbal persuasion |
| 1. Patient progress is reviewed at 6 and 12 weeks and the submaximal test is repeated (12 weeks only). Goals are reviewed and habit formation discussed. A STAMINA summary report is completed and sent to the patients clinical team at 12 weeks and a copy is provided to the patient. | Face-to-face in a consultation room | 1.1 Goal setting, 1.2 Problem solving, 1.3 Goal setting (outcome), 1.4 Action planning, 1.5 Review goal, 1.6 Review (outcome) goal, 2.1 Monitoring behaviour by others, 2.2 Feedback, 2.3 Self-monitoring, 2.7 Feedback on outcome, 3.3 Social support, 8.3 Habit formation, 10.4 Social reward, 10.9 Self reward |
| 1. Patient is encouraged to complete the STAMINA journey booklet weekly, an interactive self-monitoring tool. | Independently, booklet. | 1.1 Goal setting, 1.2 Problem solving, 1.3 Goal setting (outcome), 1.4 Action planning, 1.5 Review goal, 1.6 Review (outcome) goal, 2.3 Self-monitoring, 2.4 Self-monitoring (outcomes), 3.3 Social support, 5.1 Health consequences, 5.3 Social & environmental consequences, 5.6 Emotional consequences, 8.3 Habit formation, 9.1 Credible source, 9.2 Pros and cons, 10.9 Self reward |
