# Supplementary material for: Towards implementing exercise into the prostate cancer care pathway: development of a theory and evidence-based intervention to train community-based exercise professionals to support change in patient exercise behaviour (The STAMINA trial)
Source: BMC Health Serv Res. 2021 Mar 22;21:264. doi: 10.1186/s12913-021-06275-w (PMC7982309; doi:10.1186/s12913-021-06275-w)
Supplement: Supplementary file 5 — Additional file 5. Intervention refinements based on feedback from community-based exercise professionals and stakeholders. This file provides feedback on the intervention (training package) following rehearsal delivery to community-based exercise professionals and presentation to stakeholders. Feedback is collated into key themes. Where modifications were made, criteria for change and a description of the impact upon the intervention is provided. [file 12913_2021_6275_MOESM5_ESM.docx]

**Additional file 5: Intervention refinements based on feedback from community-based exercise professionals and stakeholders**

File 5a: A summary of feedback on the exercise professional intervention following the rehearsal delivery: mapped onto MoSCoW criteria for change

| **Key theme** | **Feedback** | **Criteria for change (MoSCoW)** | **Impact upon intervention** |
| --- | --- | --- | --- |
| Changes to the content of the training package | More pre-learning content would be useful (level 1) | Should have | - Additional information about the symptoms, prevalence and treatment of prostate cancer was added. - Community-based exercise professionals (CBEPs) will be provided with the training manual in advance of the training and directed to read the chapter on tailoring exercise for clinical populations - CBEPs will be asked to design an induction session to the gym for a man with prostate cancer on androgen deprivation therapy (ADT), who had limited gym or exercise experience. |
|  | Some video content did not play in level 1 | Must have | - Videos were broken down into multiple clips to enhance download speed and support completion of training for individuals with no access to sound. - Additional text summarising each video was added so participants still received the desired information if they experienced a technical difficulty - The automated timer between presentation slides was removed to provide unlimited time to read/ watch content. |
|  | To provide information on the practical elements of delivering the intervention, i.e. what documents to complete | Must have | - All research related documents to complete during the intervention were added to the training sessions at the relevant time point, once developed, e.g. the summary report provided in the module related to reviewing progress. |
|  | To provide a prompt sheet detailing the steps of the induction session at Nuffield Health | Should have | - Checklists detailing the content of the baseline, 6- and 12-week review were created and provided to CBEPs as prompt sheets. |
|  | To provide detailed information about ‘red flags’ and a list of key contacts for CBEPs to signpost patients to for matters beyond the scope of their role | Must have | - Additional information about spotting red flags and reporting adverse and serious adverse events was added to the exercise professional training manual - The content was also added to module 5 |
|  | To provide further detail about treatment plans and clarification of the prostate cancer care pathway | Should have | - Additional information about the prostate cancer care pathway was added to Module 1. |
|  | To consider the content of the behaviour change module. It contains large amounts of theory and role-play using skills already learnt in previous training | Must have | - Some elements of theory were simplified. - Case study examples of exercises resistance and ambivalence were added, including more advanced skills of behaviour change. - Video examples of good and bad communication were also added. - Helpful phrases in response to difficult conversations were added. |
| Recognition of completing the training package | To increase trust in the referral pathway, CBEPs should receive specialist training that is recognised/ accredited | Would like | - Accreditation of training is currently not possible until the intervention has been tested in a definitive trial. - In the meantime, CBEPs will be known as clinical exercise specialists on completion of the intervention. Completion requires a pass mark of ≥80% on two multiple choice tests (level 1) and a role play task (level 2). |
| Changes to the format of the training package | To provide more time for the interactive sessions/ tasks e.g. tailoring exercise | Should have | - The training day to allow training to be delivered both on site at the university and on the gym floor. The structure of the day was also changed due to the added flexibility. |
|  | To include more comfort breaks in the training session | Could have |  |
|  | For the ‘fitness testing’ module to be delivered on the gym floor for behavioural practise | Must have |  |

File 5b: A summary of feedback on the exercise professional training package from the stakeholder workshop: mapped onto the Normalisation Process Theory and MoSCoW criteria for change

| **Key theme** | **Feedback** | **Criteria for change (MoSCoW)** | **Impact upon intervention** |
| --- | --- | --- | --- |
| *Coherence*: Sense-making work that people do individually or collectively | Training should highlight the evidence-base to support exercise as a treatment component for men on androgen deprivation therapy | Must have | - The evidence based to support the intervention was added to the training manual. It was already included within the training package. |
| *Cognitive Participation*: Relational work that people do to build and sustain a community of practice | To increase trust in the referral pathway, community-based exercise professionals (CBEPs) should receive specialist training that is recognised/ accredited | Would like | - Accreditation of training is currently not possible until the intervention has been tested in a definitive trial. - In the meantime, CBEPs will be known as clinical exercise specialists on completion of the intervention. Completion requires a pass mark of ≥80% on two multiple choice tests (level 1) and a role play task (level 2). |
|  | Patients with prostate cancer to attend training – information from the patient voice | Would like | - Insufficient resource for men on androgen deprivation therapy (ADT) to attend training. Instead, videos of men and healthcare professionals (HCPs) discussing the symptoms, treatment and side effects of ADT were included in video format in level 1 and 2. |
| *Collective Action*: Operational work that people do to enact a set of practices | To provide detailed information about ‘red flags’ and a list of key contacts for CBEPs to signpost patients to for matters beyond the scope of their role | Should have | - Additional information about spotting red flags and reporting adverse and serious adverse events was added to the exercise professional training manual - The content was also added to module 5 |
|  | Physiologist availability may be limited. Can personal trainers be upskilled to deliver the physiologist role? | Should have | - The role of the physiologist was expanded to allow personal trainers to apply. - CBEPs must complete an application form to attend training, meeting all essential (and preferably) the desirable criteria. - Furthermore, CBEPs require a pass mark of ≥80% on two multiple choice tests (level 1) and a role play task (level 2). |
|  | All front of house staff should complete level 1 training | Must have | - Reception, brasserie and sales staff are also required to complete level 1 training. - A completion checkbox was added for notification of staff completion. |
|  | Community gyms must be provided with a deadline for completion of level 1 training | Should have | - CBEPs were provided with a deadline to complete level 1 training. Contact details were provided if they had any difficulties/ follow-up questions. |
| *Reflexive monitoring*: Appraisal work people do to assess and understand the ways a new set of practices affect them and the others around them | To upload level 1 training onto the Nuffield Health training academy for ease of access and familiarity | Would like | - The finalised intervention will be uploaded ahead of the main trial. During intervention development, level 1was hosted on a platform the research team could access for monitoring and refinement purposes. |
