# Supplementary material for: Towards implementing exercise into the prostate cancer care pathway: development of a theory and evidence-based intervention to train community-based exercise professionals to support change in patient exercise behaviour (The STAMINA trial)
Source: BMC Health Serv Res. 2021 Mar 22;21:264. doi: 10.1186/s12913-021-06275-w (PMC7982309; doi:10.1186/s12913-021-06275-w)
Supplement: Supplementary file 6 — Additional file 6. Template for Intervention Description and Replication (TIDieR) of the community-based exercise professional intervention. This file provides an overview of the community-based exercise professional intervention (training package). [file 12913_2021_6275_MOESM6_ESM.docx]

**Additional file 6: Template for Intervention Description and Replication (TIDieR) of the community-based exercise professional intervention**

| **Name** | Community-based exercise professional (CBEP) training package: Supporting exercise behaviour of men on androgen deprivation therapy (ADT) for prostate cancer |
| --- | --- |
| **Why** | The National Institute for Health Excellence (NICE) recommend that men on ADT should be offered twice weekly aerobic and resistance exercise to improve cancer specific fatigue and quality of life. However, these guidelines are not being met. CBEPs are well suited to deliver these recommendations however due to their limited training and experience of working with clinical populations, further training is required. |
| **What** | CBEPs were provided with a training manual, PowerPoint slides (online content and paper format), video examples of all behaviours, prompt sheets, case studies and associated worksheets to facilitate activities centred on tailoring exercise and writing progress reports.  **Level 1 training**  Module 1: Introduction to prostate cancer and exercise  This module provides introductory content on the symptoms, prevalence, treatments and side effects of prostate cancer and the evidence-base of exercise as a treatment component.  Module 2: Operationalising the STAMINA programme  This module provides content on scheduling patient appointments and the referral/ communication pathway between the exercise professionals and clinical team.  **Level 2 training**  Module 1: Working with clinical populations  This module focusses on developing an understanding of the target population (i.e. men with PCa on ADT). We discuss side effects, beliefs towards exercise, feelings and emotional state, previous experiences in a gym environment and demonstrate active listening skills.  Module 2: Tailoring the exercise prescription  This module covers the skills and knowledge required to tailor exercise prescriptions for clinical populations. Evidence based information is provided as well as skill-based learning with feedback.  Module 3: Delivering the exercise prescription  This module is centred on delivering tailored exercise programmes one-to-one and in small groups. We discuss the importance of monitoring exercise and practise the required skills using graded tasks and feedback.  Module 4: Reviewing the exercise prescription  This module prepares exercise professionals to review patient progress weekly and more formally at 6 and 12 weeks. Example videos are provided and exercise professionals practise completing progress reports in a role play task. Furthermore, we explore good communication skills.  Module 5: Behaviour change  This module focussed on behaviour change theory and behaviour change techniques to support initiation and maintenance of exercise, as well as ambivalence and resistance to exercise. Role play tasks are intertwined with case study examples and group discussion.  Module 6: Fitness testing  This module prepares CBEPs to conduct a submaximal fitness test. Instruction and demonstration are provided before providing exercise professionals with time to practise and receive feedback. CBEPs also practice completing the required paperwork for recording results of the fitness test and practise explaining the purpose of the test in a role play task. |
| **Who provided** | CBEPs received one full day of face-to-face training. The lead facilitator is a Behavioural Science Research Fellow with a background in Sport and Exercise Science, Psychology and Public Health. The co facilitator is the lead personal trainer from Nuffield Health with a background in biomechanics and many years of experience tailoring and supervising exercise for clinical populations. |
| **How** | The training package was delivered in two sections: online (Level 1) and face-to-face (Level 2). Level 1 was delivered individually whereas Level 2 was delivered face-to-face in small groups, ranging from 2 -10 people. |
| **Where** | The CBEP intervention was delivered on site at community exercise gyms with access to an education room (tables, chairs, projector and screen) and equipment on the gym floor for practical sessions. |
| **When and how much** | CBEPs completed level 1 and 2 once. Level 1 was administered four weeks before the scheduled level 2 training to provide sufficient time for completion. Non-completion or < 80% pass mark on the multiple-choice test in level 1, prevented CBEPs advancing to level 2 training. |
| **Tailoring** | No tailoring was required. |
| **Modifications** | Minor and major changes to the intervention were made based on feedback from CBEPs, stakeholders and a PPI group. Changes were made if they were uncontroversial and easy, repeated by several participants or deemed likely to influence behaviour. |
| **How well** | A questionnaire based on the domains of the Transtheoretical Domains Framework was developed to assess changes in CBEP behaviour pre and post intervention. |
